# Epistaxis Arrested by Subcutaneous Injections of Ergotine

**Published:** 1878-12

**Authors:** 


Epistaxis Arrested by Subcutaneous Injeccions of Ergotine.—Dr. Porak reports three cases of persistent epitaxis in each of which the hemorrhage was controlled by a single injection of ergotine under the skin. He used a solution of 2 grms. (30 grs.) of Bonjean's ergotine in 30 grms. of glycerine, and injected 20 drops into the lip or cheek.—*La Tribune Medicale.*

